# Diagnosis efficacy of CEUS for hepatic inflammatory lesions

**DOI:** 10.1002/jcla.23231

**Published:** 2020-02-03

**Authors:** Yuanyuan Guo, Xiachuan Qin, Shaoxian Chen, Xuebin Liu, Peng Gu

**Affiliations:** ^1^ Department of Gynaecology and Obstetrics Affiliated Hospital of North Sichuan Medical College Nanchong China; ^2^ Department of Ultrasound Nanchong Central Hospital Sichuan China; ^3^ Radiology Department Gaoping District People Hospital of Nanchong Nanchong China; ^4^ Department of Ultrasound Affiliated Hospital of North Sichuan Medical College Nanchong China

**Keywords:** contrast‐enhanced ultrasound imaging, differential diagnosis, hepatic inflammatory lesion

## Abstract

**Purpose:**

In this study, the efficacy of US/CEUS and clinicopathologic parameters in differential diagnosis of hepatic inflammatory lesions were evaluated.

**Methods:**

This was a retrospective study in which CEUS imaging was performed on 182 patients. Among these patients, 44 patients had hepatic inflammatory lesions and 138 patients had malignant lesions. The ultrasound (US), CEUS, and clinicopathologic parameters with respect to differential diagnosis of hepatic inflammatory lesions were analyzed.

**Results:**

Irregular lesion shape and unclear margin were commonly seen in hepatic inflammatory lesions by US/CEUS examination. Hypoenhancement in arterial phase (AP) and portal venous phase (PVP), and isoenhancement in delayed phase (DP) were more commonly found in inflammatory lesions rather than malignant lesions [9% (4/44), 68% (30/44), and 16% (7/44) vs 2% (3/138), 11% (15/138), 1% (1/138), respectively; *P* < .05]. The enhancement coverage was also a significant indicator for the differentiation of inflammatory lesions and malignant lesions (*P* < .05). History of hepatitis or cirrhosis, and higher serum alpha‐fetoprotein (AFP) level were indicators for malignant lesions, while liver parasites and higher body temperature were indicators for inflammatory lesions. When the US/CEUS findings were combined with clinicopathologic parameters, the diagnostic accuracy of inflammatory lesions could reach 93.3%, with sensitivity, specificity, positive predictive value, and negative predictive value of 63.64%, 96.03%, 84.85%, and 88.32%, respectively.

**Conclusion:**

The US/CEUS findings combined with clinical characteristics can accurately differentiate hepatic inflammatory lesions and malignant lesions. The results of study will improve the diagnostic confidence for hepatic inflammatory lesions.

## 1 INTRODUCTION

Hepatic inflammatory lesions include pyogenic liver abscess, parasitic liver abscess, inflammatory pseudotumor, and granulomatous inflammation.^1^, ^2^, ^3^, ^4^, ^5^, ^6^, ^7^ The most common symptoms are fever and abdominal pain. It usually progresses rapidly, for example, liver abscess can develop a liquefaction center within 2 weeks.^8^ However, due to the extensive use of antibiotics, hepatic inflammatory lesions usually exhibit atypical clinical features. Under the circumstances, radiological examinations often play an important role in the diagnosis of the inflammatory liver mass. Unenhanced ultrasound (US) and color Doppler ultrasonographic examination are widely used to screen liver lesions, but these techniques have limited performance in the characterization of inflammatory lesions because the inflammatory mass may present highly variable US findings depending on the pathological stage. In addition, the inflammatory lesions and malignant lesions share similar vascular pattern on color Doppler images, which may lead to misdiagnoses or even unnecessary surgery.^9^, ^10^, ^11^, ^12^, ^13^, ^14^, ^15^, ^16^, ^17^, ^18^ Contrast‐enhanced ultrasound (CEUS) with intravenous bolus injection of microbubbles can reflect tissue perfusion and improve the display of the characterization of focal liver lesions (FLL),^19^ especially for the differential diagnosis of benign and malignant FLL. CEUS is comparable to CT and MRI for the diagnosis of liver masses if an appropriate acoustic window is available.^20^, ^21^, ^22^, ^23^, ^24^, ^25^, ^26^ However, diagnostic efficacy of CEUS for the hepatic inflammatory lesions is still debatable, because it has similar perfusion pattern with malignant lesion. Therefore, in this study, differential diagnostic value of US/CEUS findings and clinicopathologic parameters for inflammatory lesions diagnosis was retrospectively analyzed to evaluate the diagnosis efficacy of CEUS for hepatic inflammatory lesions.

## 2 MATERIALS AND METHODS

### 2.1 Patients

This retrospective study involved the patients who underwent ultrasound examination at Ultrasound Department of West China Hospital and Nan Chong Central Hospital between April 2009 and February 2014. Patients were excluded if the time intensity curve (TIC) could not be drawn due to the poor quality of CEUS images, such as the patients breathed hard during arterial or late phase.

All ultrasound diagnosis was confirmed by histopathological examination of the percutaneous biopsy or surgical specimens. The study was approved by the local institutional ethics committee.

### 2.2 Clinical characteristics

The demographics, body temperature, and history of cirrhosis or biliary calculi were recorded for each patient. All the patients underwent blood routine test, including white blood cell counts (WBC), eosinophil percentage (EOS %), hepatic function tests, parasitology test, and serum tumoral marker test including alpha‐fetoprotein (AFP), carcinoembryonic antigen (CEA), and carbohydrate antigen 19.9 (CA19.9).

### 2.3 US/CEUS examination

Conventional abdominal US and CEUS examinations were performed by four US doctors who all had more than 10 years’ experiences in abdominal ultrasound and more than 5 years’ experiences in CEUS. During the conventional abdominal US examination, the size, shape, and internal echogenicity of the mass, the number of mass, and appearance of mass’ margin were recorded.

Following the B‐mode evaluation of the hepatic lesions, CEUS examinations were performed using Philips IU22 (Philips Healthcare) equipped with a C1‐5 probe. For CEUS examination, a low mechanical index (MI) was used for continuous real‐time imaging. Ultrasonic second‐generation contrast agents, sulfur hexafluoride microbubbles (SonoVue^®^), were used. An intravenous bolus of 2.4 mL SonoVue^®^ was applied followed by a bolus of 5 mL saline flush.

**Figure 1:**
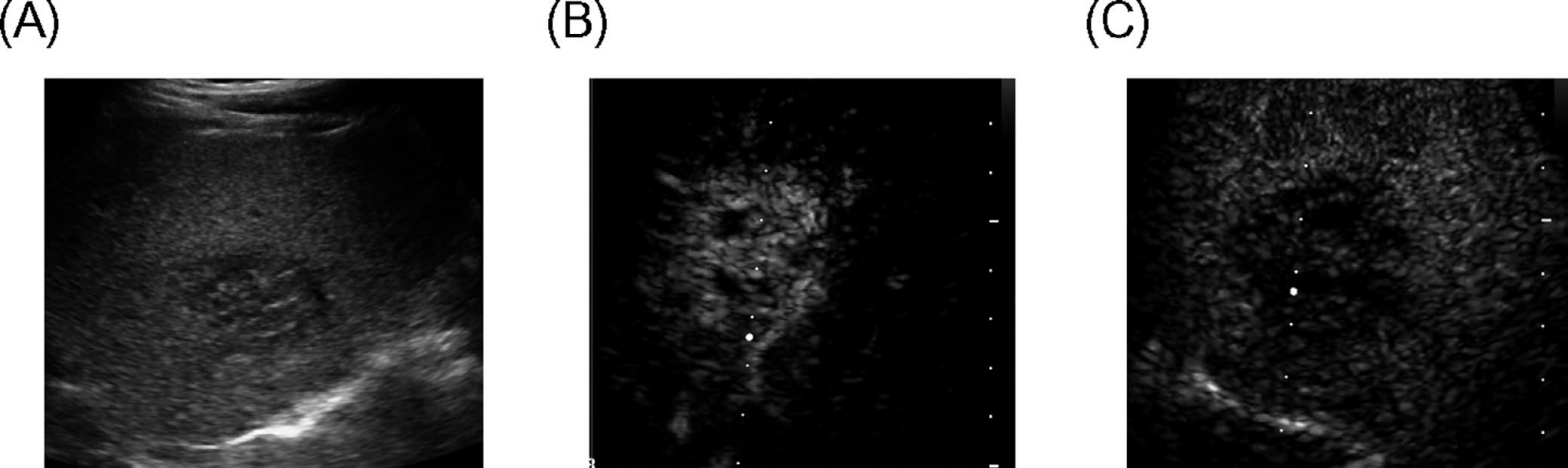
US/CEUS examination. A, Hypoechoic mass of right lobe of liver; B, the arterial phase showed rapid and high enhancement; C, the portal phase began to clear

The lesion was evaluated during three phases, 10 to 30 seconds (arterial phase, AP), 31 to 120 seconds (portal venous phase, PVP), and 121 seconds (delayed phase, DP) after SonoVue^®^ injection. The level of the enhancement of the lesion was compared to the adjacent liver parenchyma and describes as hypo‐, iso‐, or hyperenhancing (Figure 1).

### 2.4 The US/CEUS parameters’ evaluation

The US/CEUS parameters were evaluated, including (a) the maximum size of lesion (lesion size was defined as the largest diameter of the lesion under US scanning. In patients with multiple lesions, the largest lesion was selected for analysis); (b) the number of the lesions (solitary or numerous if the number of the lesions ≥2); (c) the echogenicity of the lesion (hyperechoic, isoechoic, or hypoechoic; as compared with surrounding liver parenchyma); (d) the shape of the lesion (regular or irregular); (e) the margin of the lesion (rough or smooth); and (f) the enhancement coverage, which represented the percentage of the isoenhancing or hyperenhancing area in the total area of the lesion in the arterial phase. Based on the amount of the enhancement coverage, the lesion enhancement was further categorized as following: enhancement in <50%, 50%‐75%, and 75%‐99% of lesion area and fill full (100%).

### 2.5 Statistical analysis

Age, gender, history of hepatitis and/or cirrhosis, lesion number, lesion size, baseline ultrasound echogenicity, lesion margin, lesion shape, enhancement in AP, enhancement in PVP, enhancement in DP, the enhancement coverage, WBC, serum AFP level, CEA level, CA19‐9 level, temperature, biliary calculi, parasites, and EOS% were treated as both continuous and dichotomous variables, using their respective medians for statistical analysis. Continuous variables were compared using Student's *t* test or non‐parametric Mann‐Whitney test. The diagnostic values of the clinicopathologic variables and US/CEUS findings were assessed using univariate regression analyses. The significant diagnostic factors (*P* < .05) were further subjected to a forward stepwise multivariate logistic regression to determine the independent diagnostic factors for differentiating malignant mass and inflammatory mass. All variables found to be significant on univariate analysis (*P* < .05) were entered into a step‐down Cox proportional hazard regression analysis. SPSS 10.0 software package (SPSS Inc) was used for data analysis.

## 3 RESULTS

### 3.1 Basic characteristics

A total of 182 patients were finally included, of them 44 had liver inflammatory lesions (median age: 49 years; range: 19‐75 years). Of 44 patients with liver inflammatory lesions, 16 had parasitic abscesses, 6 had inflammatory pseudotumor, 1 had granulomatous inflammation, 17 had pyogenic liver abscess, and 4 had chronic liver abscess. Malignant lesions were identified in other 138 cases (median age: 51 years; range: 26‐77 years), of whom 74 cases were diagnosed with hepatocellular carcinoma (HCC), 25 had cholangiocarcinoma carcinoma (ICC), and 39 had metastatic tumors.

### 3.2 Regression analyses of the clinicopathologic variables and US/CEUS findings

Univariate logistic regression analyses were performed to examine the diagnostic value of clinicopathologic variables and US/CEUS findings in differentiating malignant lesions and inflammatory lesions. The corresponding *P* value of each variable is listed in Table 1. Using regression analyses, 13 variables were identified, including presence of hepatitis, cirrhosis, parasites, the appearance of lesion margin, the shape of the lesion, enhancement in AP, enhancement in PVP, enhancement in DP, the enhancement coverage, WBC, AFP, body temperature, and EOS%, which were significant predictors for inflammatory lesions or malignant lesions.

**Table 1:** The univariate predictors of malignant lesions or inflammatory lesions and their corresponding P values of logistic regression analysis

Characteristic	IL	Malignant	Odds ratio	Std. Err.	*P*	95% CI
Age			0.9761882	0.0130029	.070	0.9510328‐1.002009
Sex						
Male	30	109	0.5701179	0.219639	.145	0.2679401‐1.213086
Female	14	29				
Hepatitis						
Yes	13	92	0.2119816	0.0798553	.000	0.1013079‐0.4435606
No	31	46				
Cirrhosis						
Yes	3	46	0.1463415	0.0914291	.002	0.0430096‐0.4979308
No	41	92				
US Echogenicity						
Hyperechogenicity	5	35	0.6992506	0.1628138	.124	0.4430356‐1.103639
Isoechogenicity	6	11				
Hypoechogenicity	33	92				
US Margin						
Rough	36	75	3.78	1.612533	.002	1.638222‐8.721897
Smooth	8	63				
Shape						
Regular	11	80	4.137931	1.607699	.000	1.932272‐8.861318
Irregular	33	58				
Enhancement in AP						
Hyperenhancement	37	134	0.3782785	0.1461275	.012	0.1774172‐0.8065429
Isoenhancement	3	1				
Hypoenhancement	4	3				
Enhancement in PVP						
Hyperenhancement	0	2	2.520323	0.9601924	.015	1.194444‐5.317982
Isoenhancement	14	121				
Hypoenhancement	30	15				
Enhancement in DP						
Hyperenhancement	0	0	25.9189	28.12151	.003	3.090861‐217.3471
Isoenhancement	7	1				
Hypoenhancement	37	137				
Enhancement coverage						
100%	11	52	0.5888416	0.0964398	.001	0.4271591‐0.8117219
75%‐99%	8	51				
50‐75	7	16				
<50	11	16				
Number						
Solitary	36	89	0.4670554	0.1933218	.066	0.2075115‐1.051222
Numerous	8	49				
Size(cm)			1.07442	0.0585504	.188	0.9655796‐1.19553
WBC						
Positive	17	9	7.356322	3.450993	.000	2.933226‐18.44913
Negative	27	129				
AFP (ng/mL)						
≤20	44	78	0.8910676	0.0522577	.049	0.7943117‐0.9996095
21‐400	0	35				
＞400	0	25				
CEA (ng/mL)						
Positive	6	42	0.9050758	0.0577823	.118	0.7986235‐1.025718
Negative	38	96				
CA199(U/mL)						
Positive	6	52	0.9873957	0.0074108	.091	0.9729771‐1.002028
Negative	38	86				
Temperature(℃)						
≤37.3	36	135	4.268652	1.662032	.000	1.990073‐9.156142
＞37.3	8	3				
Biliary calculi						
Positive	7	9	2.711712	1.457144	.063	0.9459075‐7.773891
Negative	37	129				
Parasites						
Positive	19	2	51.68	40.03064	.000	11.32382‐235.8587
Negative	25	136				
EOS%						
Positive	11	13	3.205128	1.455196	.010	1.316382‐7.80385
Negative	33	125				

Note

CEA > 3.4 ng/mL was considered as positive. CA19‐9 > 22 U/mL was considered as positive. WBC > 10 × 10^9^/L was considered as positive. EOS% > 5% was considered as positive.

### 3.3 Differentiating values of US/CEUS findings for inflammatory lesions and malignant lesions

Irregular lesion shape and unclear margin were more commonly found in inflammatory lesions. Irregular lesion shape and unclear margin were found in 75% (33/44) and 82% (36/44) of inflammatory lesions, respectively, whereas regular shape and smooth margin were found in 58% (80/138) and 46% (63/138) of malignant lesions, respectively (*P* < .001).

**Figure 2:**
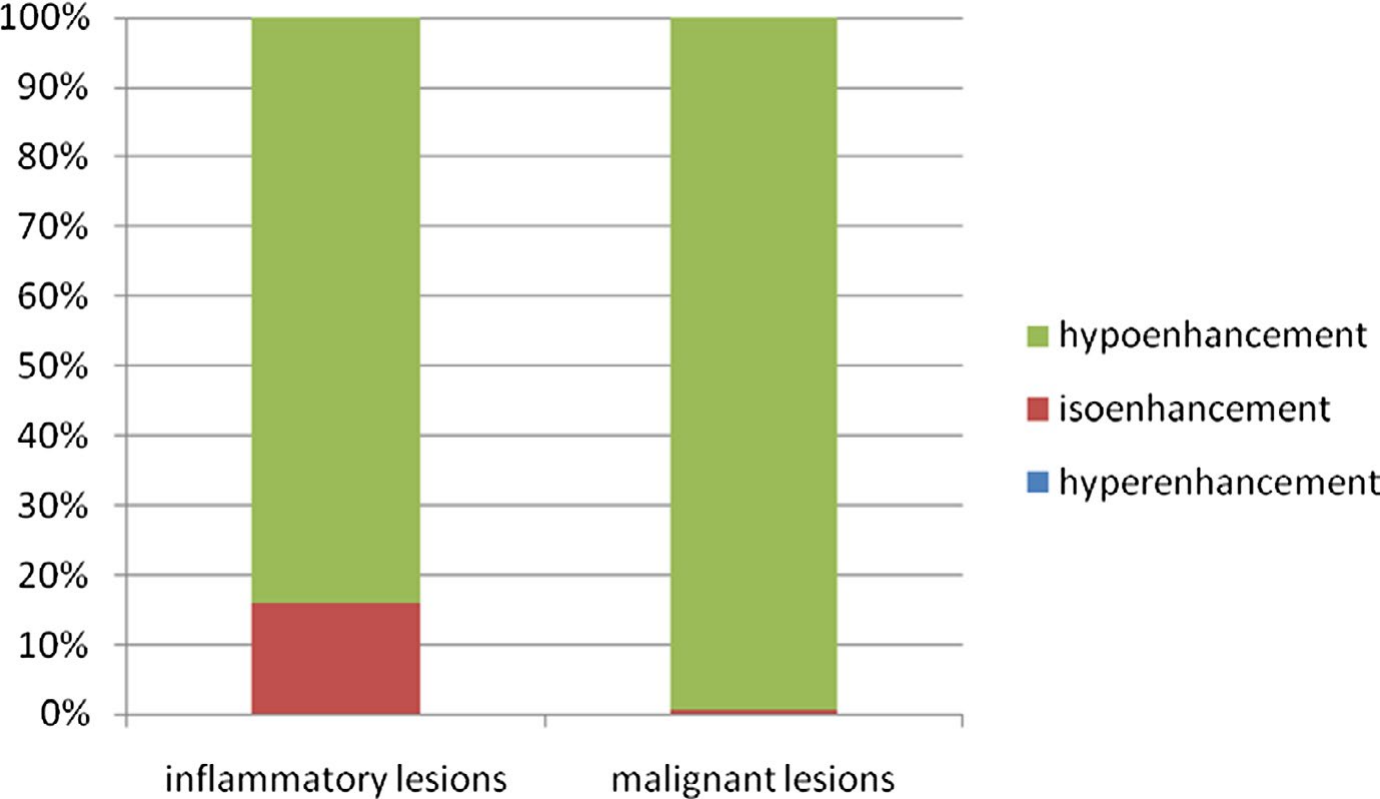
Enhancement in DP was significantly associated with malignant lesions or inflammatory lesions (*P* < .05), and 16% (7/44) of patients with isoenhancement in the DP had evidence of IL, compared to 1% (1/138) of patients with malignant lesions

The enhancement of temporal features had significant diagnostic values. Hypoenhancement in AP was more common in inflammatory lesions than in malignant lesions [9% (4/44) vs 2% (3/138), respectively; *P* < .05]. Hypoenhancement in PVP is more often seen in inflammatory lesions than in malignant lesions [68% (30/44) vs 11% (15/138), respectively; *P* < .05] (Figure 2). Enhancement in DP was significantly associated with malignant lesions or inflammatory lesions (*P* < .05). Moreover, isoenhancement in DP is more likely to be associated with inflammatory lesions than malignant lesions [16% (7/44) vs 1% (1/138), respectively; *P* < .05].

**Figure 3:**
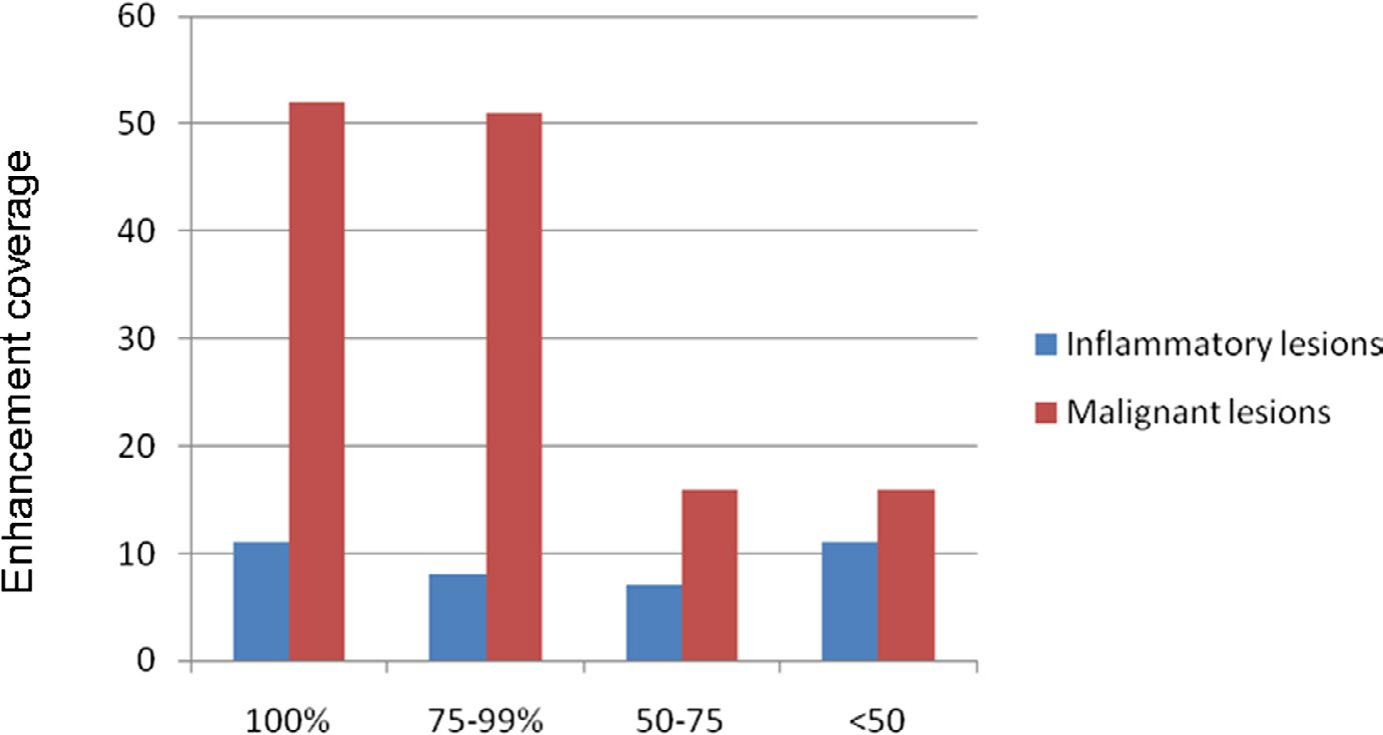
The enhancement coverage with isoenhancement or hyperenhancement during the arterial phase. The enhancement coverage has significant association with malignant lesions or inflammatory lesions (*P* < .05)

The enhancement coverage, which represented percentage area with isoenhancement or hyperenhancement in the arterial phase, was also a significant predictor for differentiating inflammatory lesions and malignant lesions (*P* < .05) (Figure 3).

**Figure 4:**
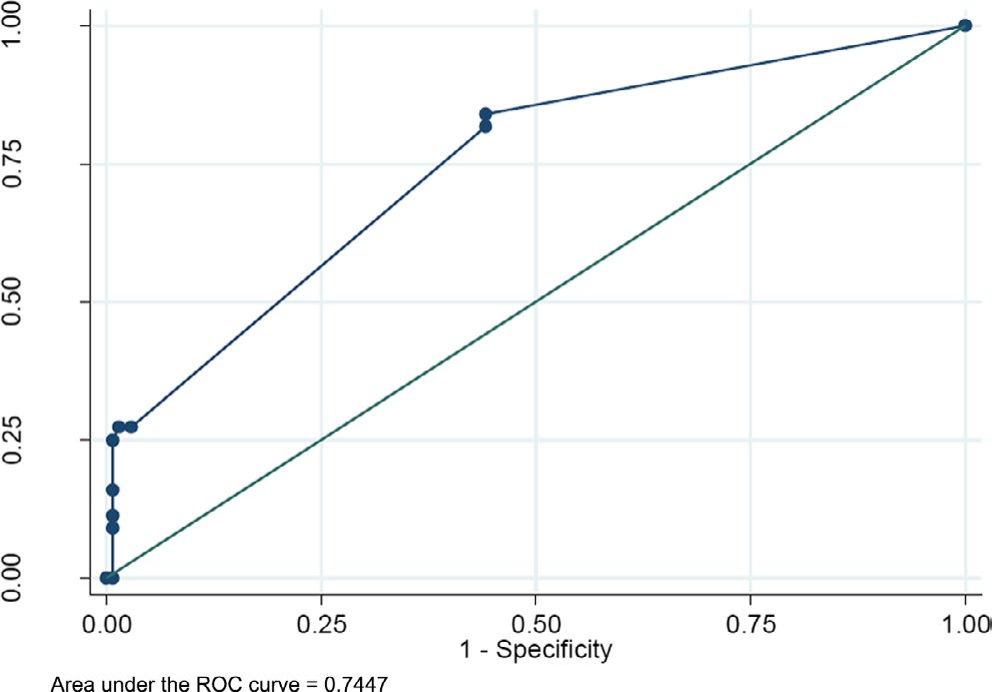
The efficiency of CEUS for IL or malignant lesions. ROC curve for the correctly diagnosed rate of CEUS. The area under the ROC curve = 0.74

The above‐mentioned significant univariate predictors were further analyzed using stepwise multivariate logistic regression model to identify the independent differential diagnostic factors for malignant lesions and inflammatory lesions. Through multi‐factor stepwise regression analysis, we identified three independent US/CEUS findings including enhancement in AP, enhancement in DP, and the shape of the lesion (Table 2). The diagnostic accuracy for IL was 74.5% based on the three independent factors as ROC curve showed (Figure 4). The sensitivity, specificity, positive predictive value, and negative predictive value of this logistic model were 27.27%, 98.55%, 85.71%, and 80.95%, respectively.

**Table 2:** Multivariate stepwise logistic regression analysis result based on univariate analysis of CEUS for malignant lesions or inflammatory lesions

Benign or malignant	Odds ratio	Std. Err.	*P *> |z|	[95% CI]
Enhancement in AP	0.4254115	0.1805614	.044	0.1851509‐0.9774457
Enhancement in DP	30.56862	34.8744	.003	3.267213‐286.0054
Shape	5.098112	2.232633	.000	2.160926‐12.0276

### 3.4 Clinicopathologic characteristics

History of hepatitis and cirrhosis was positively associated with malignant lesions. Hepatitis and cirrhosis were found in 67% (92/138) and 33% (46/138) of patients with malignant lesions, but only found in 30% (13/44) and 7% (3/44) of patients with inflammatory lesions (*P* < .05).

Liver parasitic disease and higher body temperature were associated with inflammatory lesions. Liver parasites and higher body temperature (>37.3°C) were found in 76% (19/44) and 18%（8/44）of patients with inflammatory lesions, respectively. In contrast, liver parasites and body temperature greater than 37.3°C were found in 1% (2/138) and 2% (3/138) of patients with malignant lesions (*P* < .05).

The serum AFP level of patients with malignant lesions was higher than that of patients with inflammatory lesions (*P* < .05), whereas the WBC level and EOS% level of patients with inflammatory lesions were significantly higher than that of patients with malignant lesions (*P* < .05).

The above‐mentioned significant univariate predictors were further analyzed using stepwise multivariate logistic regression model to identify independent differential diagnostic factors for malignant lesions and inflammatory lesions. Through multi‐factor stepwise regression analysis, four independent clinicopathologic parameters including AFP, temperature, parasites, and WBC were identified.

### 3.5 Diagnostic efficiency of US/CEUS findings and clinicopathologic parameters for differentiating inflammatory or malignant lesions

**Figure 5:**
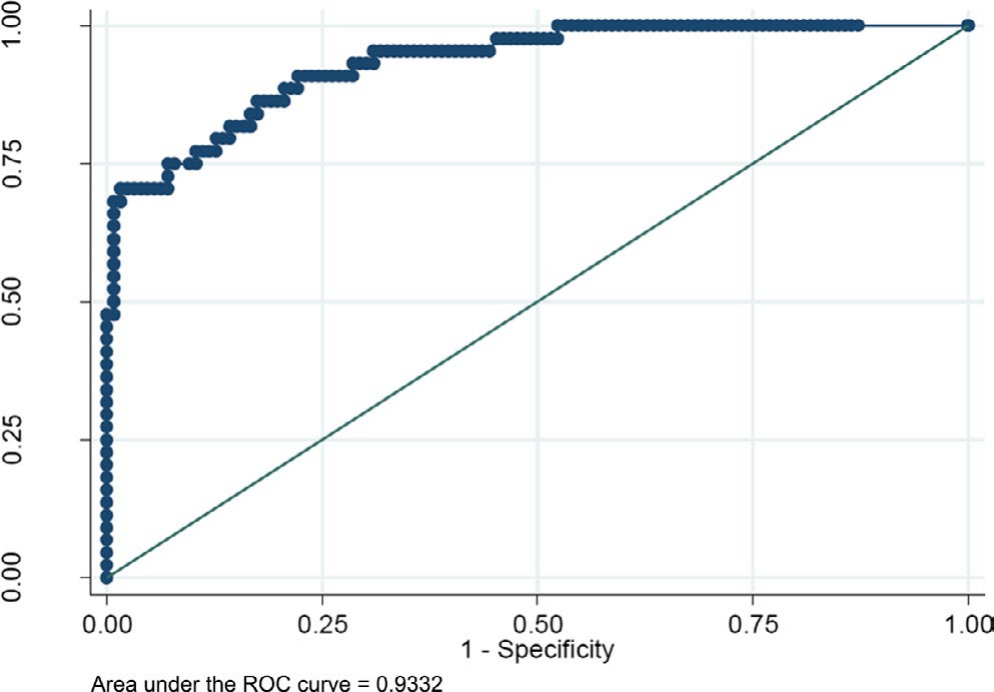
The efficiency of the five independent factors. ROC curve for the correctly diagnosed rate of the five independent factors. The area under the ROC curve = 0.93

The enhancement in AP, enhancement in DP, shape, AFP, temperature, parasites, and WBC were included in our study as independent factors. The diagnostic accuracy was 93.3% based on the independent factors as ROC curve showed (Figure 5). The sensitivity, specificity, positive predictive value, and negative predictive value of this logistic model were 63.64%, 96.03%, 84.85%, and 88.32%, respectively.

## 4 DISCUSSION

CEUS showed exquisite vascularity and tissue perfusion in real‐time and excellent spatial resolution.^27^ In this study, we performed CEUS in two cohorts of patients, analyzed the temporal features of enhancement in AP, PVP, and DP, and investigated the differential diagnostic value of the CEUS‐derived parameters in liver inflammatory lesion and malignant lesions. Most malignant lesions showed hyperenhancement during the arterial phase, with hypoenhancement or isoenhancement in the portal venous and late phase. ICC and hepatic metastases often showed a rim‐like hyper‐/isoenhancement with the enhancement coverage less than 50% in AP, followed by hypo‐/isoenhancement during the PVP and LP.^28^, ^29^, ^30^ The rim‐like enhancement pattern also appeared in the inflammatory lesion due to the formation of necrotic center or peripheral inflammatory cell infiltration and granulation tissues. Although the pathological changes of inflammatory lesions vary, the distribution of the enhancement within all inflammatory lesions was similar. The enhancement pattern of inflammatory lesions in AP was nonspecific.

Washout enhancement pattern was considered as the presence of hypoenhancement of the lesion in the portal or late phases preceded by hyperenhancement in the arterial phase. Lesions with washout enhancement pattern should be considered as malignant until proven benign.^31^ However, in our study, it was found that the proportion of patients with isoenhancement in DP was more likely to be diagnosed with hepatic malignant tumor. When the lesion showed isoenhancement in DP, it obviously increased the confidence of IL. We could find 84% (37/44) typical of IL showed hypoenhancement in DP. The proportion of IL, which showed hypoenhancement in DP, was more than the malignant lesions, which meant that the IL washout was faster than the malignant lesion. Washout enhancement in DP was more common in inflammatory lesions [84% (37/44)] than that in malignant lesions in this study, which was consistent with previous research^18^ and suggested that inflammatory lesions washout was faster than the malignant lesions. Nonetheless, washout enhancement pattern could not be used as an independent factor for differential diagnosis of inflammatory lesion and malignant lesion. Inflammatory lesions were more likely to have irregular shape because of chronic inflammation in the lesion and the surrounding tissue. We identified three independent US/CEUS‐derived parameters using stepwise multivariate logistic regression model. The area under the ROC curve of the CEUS judgment was 0.73, which meant it was useful for differentiating inflammatory and malignant lesion. In addition, the US/CEUS‐derived parameters could not distinguish inflammatory lesions and malignant lesions independently. It is necessary to take into account both the patient's medical history and laboratory tests.

Cirrhosis existed in both HCC and ICC,^32^, ^33^, ^41^ while chronic viral hepatitis has been recognized as the most important risk factor for cirrhosis development.^34^, ^35^, ^36^ In addition to chronic viral hepatitis, hepatic parasitic diseases also lead to cirrhosis.^37^, ^38^, ^39^, ^40^ Although not all HCC and especially ICC cases have been recognized as risk factors, primary sclerosing cholangitis,^42^ hepatobiliary flukes,^43^ intrahepatic stones,^44^ and biliary tract malformation have been considered to have the ability to increase the incidence of ICC. Most hepatic inflammatory lesions originated from the liver,^7^, ^50^ but they could also show only extrahepatic symptoms.^45^, ^46^, ^47^, ^48^, ^49^ Overall, the etiologies of hepatic malignancies and inflammatory lesions vary, and these risk factors often mingled with each other and made it difficult for accurate diagnosis if we only consider some of the factors. Therefore, it is reasonable to bring in more clinicopathological parameters to improve the diagnosis accuracy.

Tumor markers are specific antigen used as a biomarker of malignant cellular transformation.^51^, ^52^, ^53^, ^54^, ^55^ We included the clinically widely used tumor serum markers including AFP, CEA, and CA199 in this study and aimed to investigate whether tumor marker measurement would facilitate differential diagnosis of inflammatory lesion and malignant lesion. AFP was not found to be a significant factor as suggested by multivariable analysis; CEA and CA199 might be taken into account. Although chills and fever are not always typical in inflammatory lesions because of the extensive use of antibiotics, we found that higher body temperature indicated inflammatory lesions, especially when combined with parasites and WBC tests. In this study, liver inflammatory lesions had rapid enhancement in the arterial phase and washout in the portal venous or the late phase, which is nonspecific and well‐resembled malignant lesions. Therefore, CEUS‐derived temporal enhancement parameters could not be solely relied on for differential diagnosis of inflammatory lesion and malignant lesion. However, when combined with patient medical history and laboratory test, the diagnosis confidence was greatly improved.

The limitation to our study is that the number of patients with inflammatory lesions was small while that of patients with malignant lesions was large. Further research with larger numbers was needed.

In conclusion, the CEUS‐derived temporal parameters combined with clinical characteristics can accurately differentiate hepatic inflammatory lesions and malignant lesions. Our method might be a potential way to improve the diagnostic confidence for hepatic inflammatory lesions.
